# Evaluation of therapeutic use of a combination of pentoxifylline and vitamin E in radiation-induced renal fibrosis

**DOI:** 10.1038/s41598-024-57850-0

**Published:** 2024-03-23

**Authors:** Volkan Demircan, Caglar Guzel, Gulistan Sanem Sarıbas, Serap Catlı Dinc, Serhat Cetin, Ozlem Gulbahar, Petek Erpolat, Cigdem Elmas, Huseyin Bora

**Affiliations:** 1 Radiation Oncology Department, Acibadem Altunizade/Atasehir Hospital, Altunizade Mah. Sehit Erol Olcok Sok. No:7 Albayrak Sitesi B Blok Daire:18, Uskudar/Istanbul, Turkey; 2 Radiation Oncology Department, Gazi University Medical Faculty, Ankara, Turkey; 3 Histology and Embryology Department, Health Sciences University Gulhane Medical Faculty, Ankara, Turkey; 4 Urology Department, Gazi University Medical Faculty, Ankara, Turkey; 5 Biochemistry Department, Gazi University Medical Faculty, Ankara, Turkey; 6 Histology Department, Gazi University Medical Faculty, Ankara, Turkey

**Keywords:** Medical research, Preclinical research

## Abstract

Radiation-induced renal fibrosis (RIRF) is a progressive, irreversible condition causing chronic kidney disease. Pentoxifylline (PTX) and vitamin E may mitigate radiation-induced damage and fibrosis. This study assesses their effectiveness. We used four groups, each with six rats: radiation therapy alone (RT-only), radiation therapy plus drug treatment (RT + drug), drug treatment alone (drug-only), and a control group. Rats were monitored for three months, with weight measurements every four weeks. Afterward, rats were analyzed biochemically and histologically, with blood and tissue samples taken for statistical comparison. No significant differences in serum creatinine levels and body weight were observed. RT-only group had more severe kidney tubule effects. Histomorphological, immunohistochemical, and TUNEL analyses showed significant RIRF mitigation in the RT + drug group. Our study highlighted molecular pathways (SMAD, TGF-beta, VEGF) and histological markers (collagens, a-SMA, fibronectin, metalloproteinases) associated with RIRF. PTX and vitamin E reduced ionizing radiation's impact on renal cells and mitigated radiation-induced kidney fibrosis. Further human studies are needed to confirm these findings.

## Introduction

The kidneys are routinely exposed to radiation during radiotherapy (RT) for gastrointestinal, gynecological, and genitourinary malignancies, as well as total body irradiation (TBI), due to their proximity to or inclusion within the treatment field, establishing them as substantial dose-limiting organs. Exposure to irradiation often causes acute glomerular and tubular damage, which advances to chronic tubulointerstitial fibrosis^1^. Consequently, chronic kidney disease (CKD) can develop, with a long-term incidence rate of up to 50% in TBI patients^2^. CKD has a remarkable impact on quality of life and a poor prognosis, with a 5-yr survival rate ranging from 25 to 35%^3^.

Previous studies have revealed the effects of radiation on gene expression, particularly in the context of extracellular matrix (ECM) protein synthesis or degradation inside rat renal tubular and mesangial cells^4^. When ECM proteins are activated or inhibited, fibroblasts transition into myofibroblasts, causing the renal interstitium to expand, peritubular capillary vessels to be lost, and tubular structure to be disrupted, ultimately resulting in renal fibrosis^5^. In conclusion, intracellular oxidative mechanisms and the TGF-β as well as mTOR pathways play critical roles in renal fibrosis. Target molecules, such as TGF-α, TGF-β, and TNF-α, have been shown to remodel the cell cytoskeleton (vimentin, α-SMA) via metalloproteinase (MMP) activity throughout this process via intermediary molecules, such as SMAD and NF-κB^6^. A previous study has shown that radiation-induced renal fibrosis (RIRF) was observed in the 10- to 20-Gy dose range after the second month and these effects increase with time and dose^7^.

Pentoxifylline, a vasodilatory agent known to improve tissue oxygenation and nutrition, has shown therapeutic potential. Its inhibitory effects on TNF-α, IL-1, FGF, TGF-β, and the SMAD pathway, along with its ability to reduce fibroblast activity and inflammation, make it an effective therapeutic agent^8,9^. These properties have prompted promising preclinical studies of pentoxifylline for treating kidney fibrosis caused by ureteral obstruction or using contrast agents^10,11^. Furthermore, it has been shown to protect against tubulointerstitial fibrosis while increasing VEGF expression^12^.

Vitamin E, a tocopherol family fat-soluble antioxidant, protects against the damaging effects of free radicals. The nitric oxide synthase enzyme preserves cell membrane fluidity and has vascular-protective, anti-inflammatory, and antifibrotic properties^13^. Furthermore, it has been shown to prevent fibrosis and apoptosis via its influence on TGF-β, which calls for future research into its therapeutic effects on renal fibrosis, similar to pentoxifylline^14^.

Intriguingly, the therapeutic potential of a pentoxifylline and vitamin E combination regimen has been investigated in clinical and preclinical settings for a variety of indications, including brain necrosis, skin fibrosis, pulmonary fibrosis, and radiotherapy-induced osteoradionecrosis^15–19^. However, data on the efficacy of this combination in treating RIRF is scarce. Given the evident relationship between the mechanisms of action of this drug combination and the pathogenesis of RIRF, a focused study is warranted. Therefore, we conducted a study using 24 Wistar rats to investigate the effects of combining pentoxifylline and vitamin E on radiation therapy-induced renal fibrosis.

## Materials and methods

### Experimental setting and animal care

This study was carried out at the Gazi University Laboratory Animal Breeding and Experimental Research Centre. We used 24 adult male Wistar rats, weighing between 150 and 300g. Rats were housed in cages with groups of six kept under standard conditions with a 12 h light–dark cycle and granted ad libitum access to food. The study design was shown in Table 1.

**Table 1:** Study design.

Groups	RT (single dose)	Pentoxifylline Protocol (50 mg/kg/day)	Vitamin E Protocol (5.5 mg/kg/day)
1.group (RT + drug) (treatment group)	15 Gy	From the first day of the experiment, 50 mg/kg/day , via oral gavage	From the first day of the experiment 5.5 mg/kg/day, via oral gavage (dissolved in 0.1 ml olive oil)
2. group (RT only)	15 Gy	–	–
3. group (Drug only)	–	From the first day of the experiment, 50 mg/kg/day , via oral gavage	From the first day of the experiment 5.5 mg/kg/day, via oral gavage (dissolved in 0.1 ml olive oil)
4. group (control)	–	–	–

*The experiment duration time was 12 weeks for all groups.

**The rat number of all groups was 6.

### Drug administration

Both pentoxifylline and vitamin E were administered orally to rats. Based on the existing literature, the dose of pentoxifylline was established at 50 mg/kg/day and administered by oral gavage. Similarly, vitamin E was administered at a dose of 5.5 mg / kg / day, according to the recommendations of the literature^20^. Before administration, vitamin E was dissolved in 0.1 ml of olive oil. For the cohort undergoing radiation therapy (RT), drug administration began the day after RT initiation and persisted for the subsequent 12 weeks, culminating in the euthanasia of the rats.

### Radiotherapy procedure

**Figure 1:**
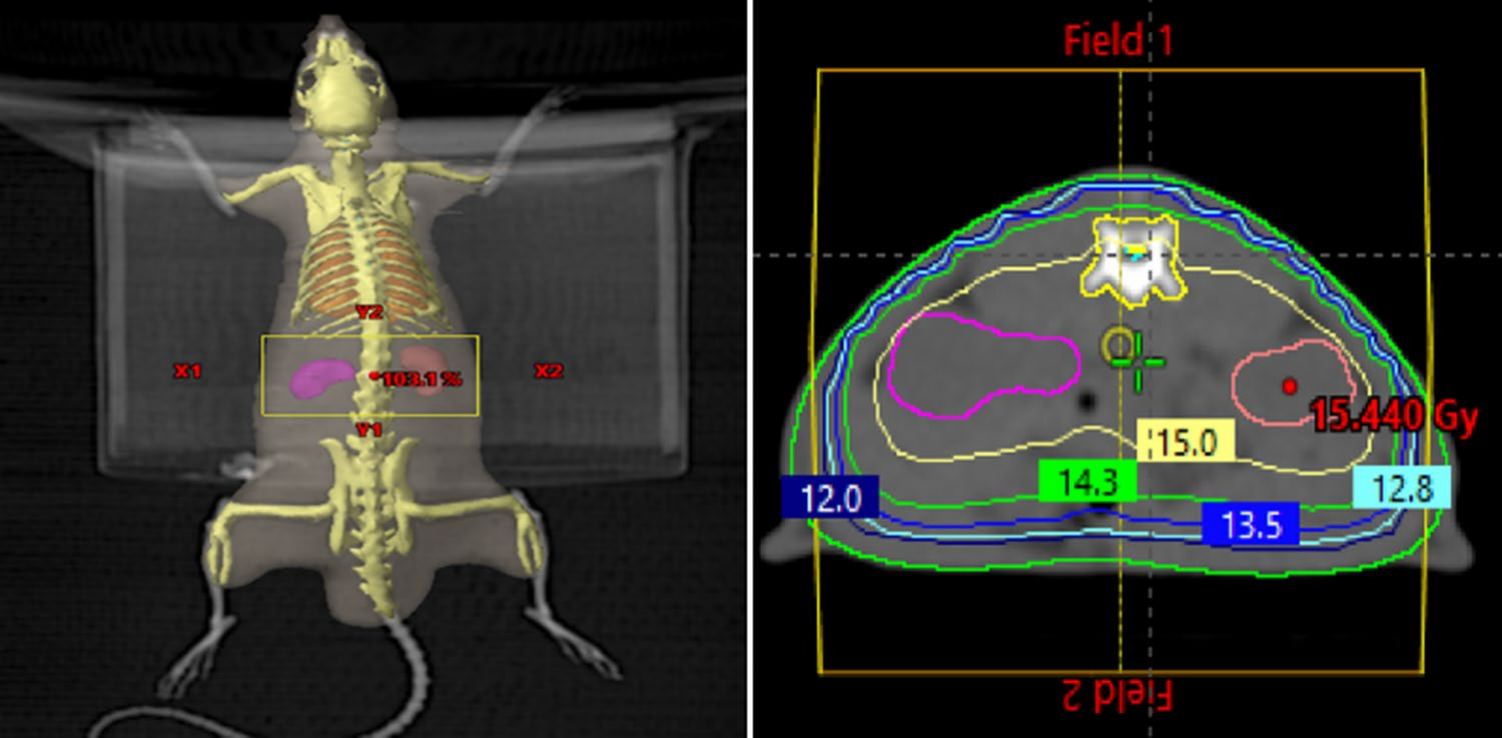
(**A**) Bilateral kidneys were irradiated with anteroposterior (AP) and posteroanterior (PA) fields. (**B**) Dose distribution on CT images of rats. Fifteen gray isodose line covers the both of the kidneys.

The rats in groups 1 and 2, received an intramuscular injection of 50 mg / kg of ketamine and 5 mg / kg of xylazine for immobilization. Once sedated, the animals were positioned supine to facilitate computerized tomography (CT) imaging for simulation purposes. Based on these CT scans, a three-dimensional conformal RT (3D-CRT) plan was devised to encompass both kidneys. Subsequently, a single dose of 15 Gy RT was delivered using a linear accelerator (Fig. 1).

### Study duration and analysis

The experimental timeline spanned 12 weeks, a duration selected in alignment with the anticipated period for the manifestation of radiation-induced renal fibrosis^7^. Weight assessments were performed at intervals of 1, 4, 8, and 12 weeks before euthanasia. Upon sacrifice, both kidneys were extracted and any macroscopic variations in color and texture were meticulously documented.

In the evaluation of histological findings scoring, observations included hemorrhage, tubular necrosis, mononuclear cell infiltration, vascular dilation, tubular epithelial cell degeneration, glomerular degeneration, Bowman's capsule degenerations, and hydropic changes. Each section from the groups stained with H&E was examined in six different areas. The results were assigned scores based on the degree of damage as follows: 0 = no damage, 1 =  < 50% damage (focal), 2 =  > 50% damage (diffuse).

### Immunohistochemical method

For immunohistochemical evaluations, coronal sections, each 4 µm thick, were prepared. In addition to standard Hematoxylin–Eosin staining to discern fibrotic regions, immunohistochemical assays were performed to determine levels of VEGF, alpha-SMA, TGF-beta, MMP9, TIMP1, fibronectin, collagen 1 and 4, SMAD, and CTGF antibodies.

Paraffin blocks were cut into 4 μm thick sections for immunohistochemical staining and mounted on polylysine-coated slides. The sections underwent an overnight incubation at 37° C and were subsequently heated to 57° C for one hour, followed by 61° C for 20 min, to advance deparaffinization. The process was concluded with two 20 min xylene immersions each. Alcohol solutions of progressively decreasing concentrations were used for dehydration of the sections, which were then twice rinsed in distilled water for alcohol removal. The sections were exposed to citrate buffer (pH 6.0) in a microwave oven to uncover formaldehyde-obscured receptor regions.

Following a cooling period of 20 min and subsequent washes with distilled water, the tissues were enclosed by a PAP pen and placed within a humid immunohistochemical chamber. After triple rinses with PBS, endogenous peroxidase activity was blocked by a 15-min incubation in 3% hydrogen peroxide, followed by washes with PBS. Nonspecific binding was prevented by 5 min of UltraV block application and sections were incubated overnight at + 4 °C with primary antibodies (Fibronectin (bs-0666R, rabbit polyclonal, Bioss Inc.), TGFβ1 (bs-0086R, rabbit polyclonal, Bioss Inc.), Collagen-1 (bs-10423R, rabbit polyclonal, Bioss Inc.), Collagen-4 (bs-4595R, rabbit polyclonal, Bioss Inc.), p-SMAD3 (bs-3425R, rabbit polyclonal, Bioss Inc.), CTGF (bs-0743R, rabbit polyclonal, Bioss Inc.), αSMA (bsm-52392R, rabbit monoclonal, Bioss Inc.), VEGF (bs-1665R, rabbit polyclonal, Bioss Inc.), MMP9 (bs-0397R, rabbit polyclonal, Bioss Inc.) ve TIMP1 (bs-0415R, rabbit polyclonal, Bioss Inc.) at a 1: 200 dilution. After this incubation, the sections were rinsed three times with PBS and a biotinylated secondary antibody was applied for 10 min. After the PBS wash, the sections underwent a 10-min treatment with a streptavidin-peroxidase enzyme complex, followed by triple PBS washes. The substrate diaminobenzidine (DAB) was then added and incubation ensued until the immunological reaction was observable under a microscope. Mayer's hematoxylin was used for background staining. After DAB labelling and background staining, the slides were subjected to a dehydration process, followed by entellan application and coverlip attachment. Computer-assisted imaging was used to photograph and evaluate the sections, and immunohistochemical binding densities were determined using ImageJ software for each primary antibody. For each subject, these ratios were randomly calculated in six different areas under 200X magnification using the ImageJ programme.

### TUNEL method

The TUNEL method was used to examine apoptotic bodies related to fibrosis.

The Apo-BrdU-IHC method (TUNEL-Terminal deoxynucleotidyl transferase dUTP nick end labeling) method was employed to detect apoptosis via DNA fragmentation. Sections of 4-5μm thickness on slides were deparaffinized by incubation at 61° C for one hour, according to the BioVision ApoBrdU-IHC DNA fragmentation assay kit (cat no: K403) protocol, and further exposed to xylene twice for 10 min each. Sequential immersion in series of alcohol for 5 min each led to dehydration. The sections were then washed with 1X PBS and incubated with Proteinase K at room temperature for 20 min. The tissues were then exposed to 3% H2O2 for 5 min, washed with PBS, and incubated with 1X reaction buffer for 20 min at room temperature. After the removal of the buffer, the tissues were incubated with DNA labelling solution for 1.5 h at 37 °C in a humid environment. After washing, tissues were incubated with antibody solution for 1.5 h, after blocking for 10 min. The antibody solution was then removed and the tissues were covered with blocking buffer, followed by application of 1X conjugate solution for 30 min. After washing, DAB solution was applied and the reaction was monitored under a microscope for 15 min. A methyl green solution was used for background staining. After dehydration, the sections were sealed with entellan, photographed, and evaluated with a computer-assisted imaging system. Quantification of TUNEL-positive cells was performed through the analysis of six randomly chosen regions at 400 × magnification with Image J.

### Biochemical analysis

Serum creatinine levels were biochemically assayed in two steps: initially using blood samples extracted from the rats' tail veins before the experiment and subsequently from intracardiac sites after the experiment.

Rat serum samples for creatinine determination were collected in gel tubes with yellow caps and left at room temperature for 20 min, followed by centrifugation at 3000 rpm for 20 min. The serum obtained was then stored in Eppendorf tubes at −80 °C until analysed using a commercial ELISA kit (Shanghai Sunred Biological Technology Co., Ltd) using the ELISA method. The washing process in the ELISA analysis was performed using a Biotek washer (ELx50 Bioelisa Washer, Bio-Tec. Instruments, Inc.), and absorbance readings were recorded using a Biotek reader (ELx800 UV Universal Microplate Reader, Bio-Tec Instruments, Inc.).

### Statistical methods

The normality of continuous variables was evaluated graphically using the Shapiro–Wilk test. Descriptive statistics were provided as mean ± standard deviation, median and minimum and maximum values for continuous data, and as percentages for categorical variables (histopathological damage scoring). One-way analysis of variance (ANOVA) was used for the comparison of parametric variables, with the Bonferroni post hoc test for source identification when a difference was detected. Nonparametric data was compared using Kruskal–Wallis nonparametric analysis of variance, and pairwise comparisons were made using the Bonferroni corrected Mann–Whitney test if a difference was detected. The Pearson's chi-square test was used to analyse differences between ordinal-categorical variables. All statistical analyses and computations were performed using IBM SPSS Statistics version 21 (IBM Corp, NA, USA). A *p*-value ≤ 0.05 was considered significant.

### Ethical considerations

Ethical clearance was obtained from the Gazi University Experimental Animal Research Ethics Committee on 26.06.2020 with code number of G.U.ET 20.010.

All experimental protocols received approval from the Gazi University Experimental Animal Research Ethics Committee. All methods were conducted in strict adherence to relevant guidelines and regulations. Group sizes were determined based on the minimum number required for proper statistical analysis. Throughout the experiment, all authors strictly avoided any unethical behavior towards the animals. All procedures were executed smoothly, ensuring that the animals did not experience stress.

Furthermore, all methods have been reported in accordance with the ARRIVE guidelines.

## Results

### Rat weights

**Figure 2:**
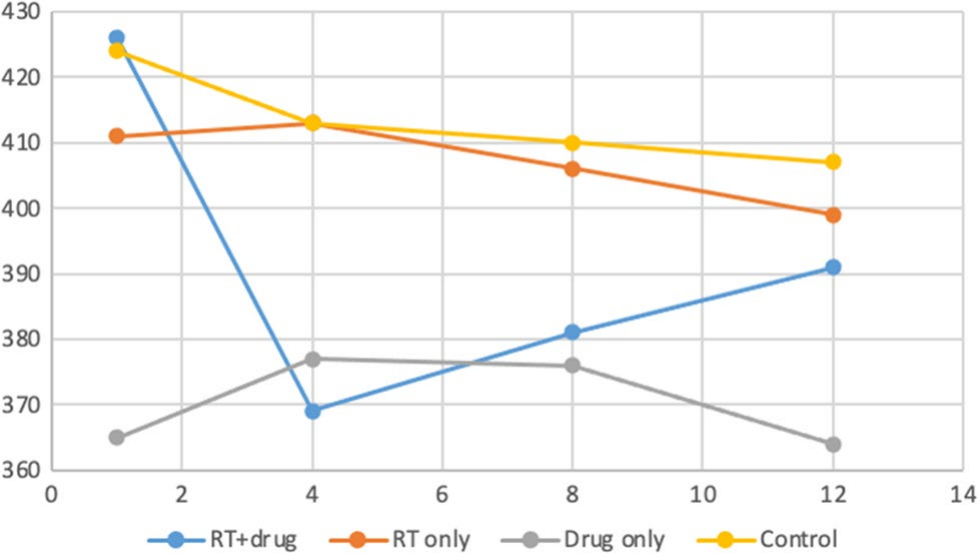
Comparison of weights within and between groups (Vertical axis indicates weights and horizontal axis indicates weeks) No comparable differences were observed in the RT-only (*p* = 0.665), drug-only (*p* = 0.858), and control groups (*p* = 0.133). However, within the RT + drug cohort, significant statistical differences were observed in the change in median weights over time (*p* < 0.001).

The median weight values among the groups at week one (*p* = 0.077), week four (*p* = 0.159), week eight (*p* = 0.271), and week twelve (*p* = 0.374) exhibited no statistically significant variation. However, within the RT + drug cohort, significant statistical differences were observed in the change in median weights over time (*p* < 0.001). This variance mainly originated from the differences between week one, four, and twelve. The median weight values of the RT + drug group for the first, fourth, eighth, and 12th weeks were 443.5g, 366g, 379g, and 387g, respectively. No comparable differences were observed in the RT-only (*p* = 0.665), drug-only (*p* = 0.858), and control groups (*p* = 0.133) (Fig. 2) (Table 2).

**Table 2:** Comparison of intra-group and inter-group weights of rats.

	RT + Drug	RT only	Drug only	Control	Test stats	p*
1st week weight	426 ± 45.75	410.83 ± 29.55	365 ± 41.4	424.17 ± 66.17	6.838	0.077
	443.5 (339–460)^a^	399 (385–448)	356 (324–425)	444.5 (296–480)		
4th week weight	368.5 ± 45.94	413 ± 32.05	377.17 ± 46.12	413 ± 71.17	5.183	0.159
	366 (300–423)^b^	406 (374–468)	375 (320–444)	438.5 (273–462)		
8th week weight	381.33 ± 48.43	406.33 ± 55.26	376.17 ± 38.46	410 ± 74.92	3.913	0.271
	379 (312–446)^ab^	411 (310–469)	374.5 (328–437)	435.5 (265–466)		
12th week weight	391.17 ± 49.48	398.67 ± 76.43	363.67 ± 42.62	407.17 ± 79.35	3.116	0.374
	387 (321–460)^a^	416.5 (256–470)	360 (307–430)	433.5 (256–470)		
Test stats	17.746	1.576	0.763	5.600		
p**	** < 0.001**	0.665	0.858	0.133		

*Kruskall Wallis H test, **Friedman test, mean ± s. Standard deviation, median (min – max), a-b: There is no difference between times with the same letter.

Significant values are in bold.

### Creatinine levels and rat weights

No statistically significant associations were found between creatinine levels and weight within the RT + drug, RT-only, drug-only, and control groups at weeks one, four, eight, and twelve (Table 3).

**Table 3:** Comparison of creatinine values according to groups.

	Mean ± SD	Median (min.–max.)	Test statistics	p*
RT + drug	122.17 ± 23.26	120.5 (91–155)	3.407	0.333
RT only	144.5 ± 34.54	146 (99–197)		
Drug only	138.5 ± 29.29	122.5 (113–178)		
Control	151.33 ± 27.35	161.5 (107–174)		

*Kruskall Wallis H test, RT: Radiotherapy, SD: Standard deviation.

There was no statistically significant difference found among creatinine values according to groups (*p* = 0,333).

### Histomorphological findings

**Figure 3:**
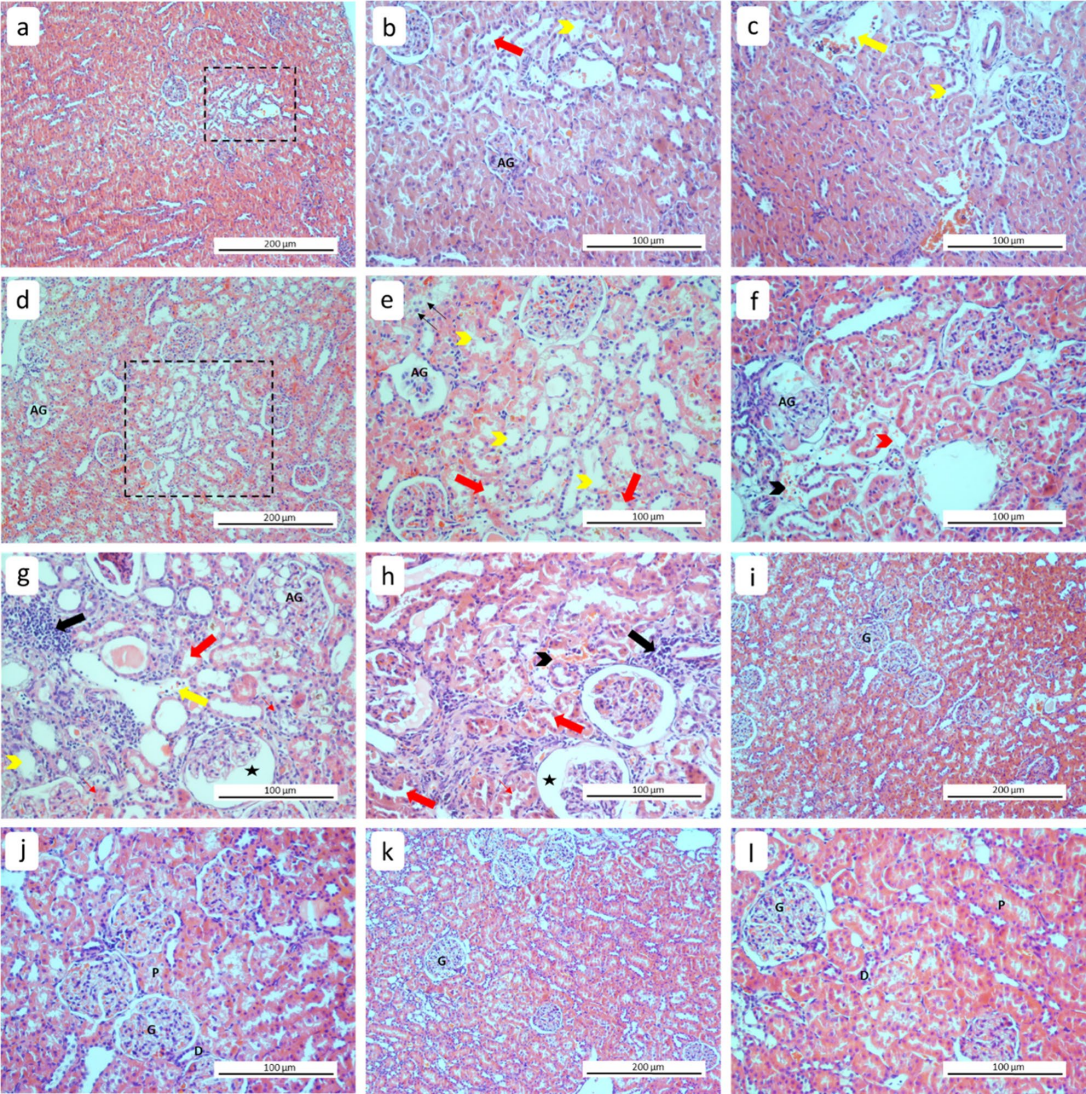
Representative histological images of kidney tissues from experimental groups. (**a**–**c**) RT + Drug (treatment), (**d**–**h**) RT, (**i**–**j**) Drug, (**k**–**l**) control. Tubular necrosis (striped square area), hemorrhage (black arrowhead), mononuclear cell infiltration (thick black arrow), vascular dilation (yellow arrow), Bowman's space dilation (star), hydrophilic degeneration (red arrowhead), atypical dilation in tubular cells (thin black arrow), brush border loss (thick red arrow), cellular debris (thin red arrow), dilated tubules (yellow arrowhead), G: normal glomerulus, AG: atypical glomerulus, D: distal tubule, P: proximal tubule (H&E).

In the RT + drug group, nodular tubular necrosis, non-dense atypical glomerular formations, vascular dilation and loss of microvillus were observed, other renal structures similar to the control group (Fig. 3a–c).

The RT-only group showed diffuse tubular necrosis, haemorrhage, mononuclear cell infiltrations, vascular dilations, atypical epithelial cell dilations, and intertubular hydrophobic degeneration (Fig. 3d–h). On the contrary, kidney structures within the drug-only (Fig. 3i–j) and control (Fig. 3k–l) groups showed normal histological findings.

Histopathological scoring did not show significant differences between the control and drug-only groups (*p* = 0.743). The RT-only and treatment groups, compared to the control, showed a statistically significant increase in renal damage (*p* < 0.001). However, renal damage was less severe in the treatment group compared to the RT-only group (*p* = 0.012) (Tables 4 and 5).

**Table 4:** Histopathological damage score.

SCORE	Histopathological scoring (%)			
	1.group	2.group	3.group	4.group
0	22.2	5.6	83.3	86.1
1	41.7	25	16.7	13.9
2	36.1	69.4	–	–
total	100	100	100	100

Score: 0: no damage, 1: < 50% (focal), 2: > 50% (diffuse).

**Table 5:** The pairwise comparison of groups for histopathological damage scoring.

Groups	*p*-value*
Control vs. Drug	0.743
Control vs. RT	** < 0.001**
Control vs. RT + drug	** < 0.001**
RT vs. RT + drug	**0.012**
RT vs. Drug	** < 0.001**
RT + drug vs. Drug	** < 0.001**

Significant values are indicated in bold. * Chi-square (Pearson) test.

### Immunohistochemical evaluation

Immunohistochemical evaluations included calculating the positive staining ratio of antibodies against various markers, including fibronectin (FN), TGFβ1, Collagen-1 (COL1), Collagen-4 (COL4), p-SMAD3, CTGF, αSMA, VEGF, MMP9, and TIMP1.

**Figure 4:**
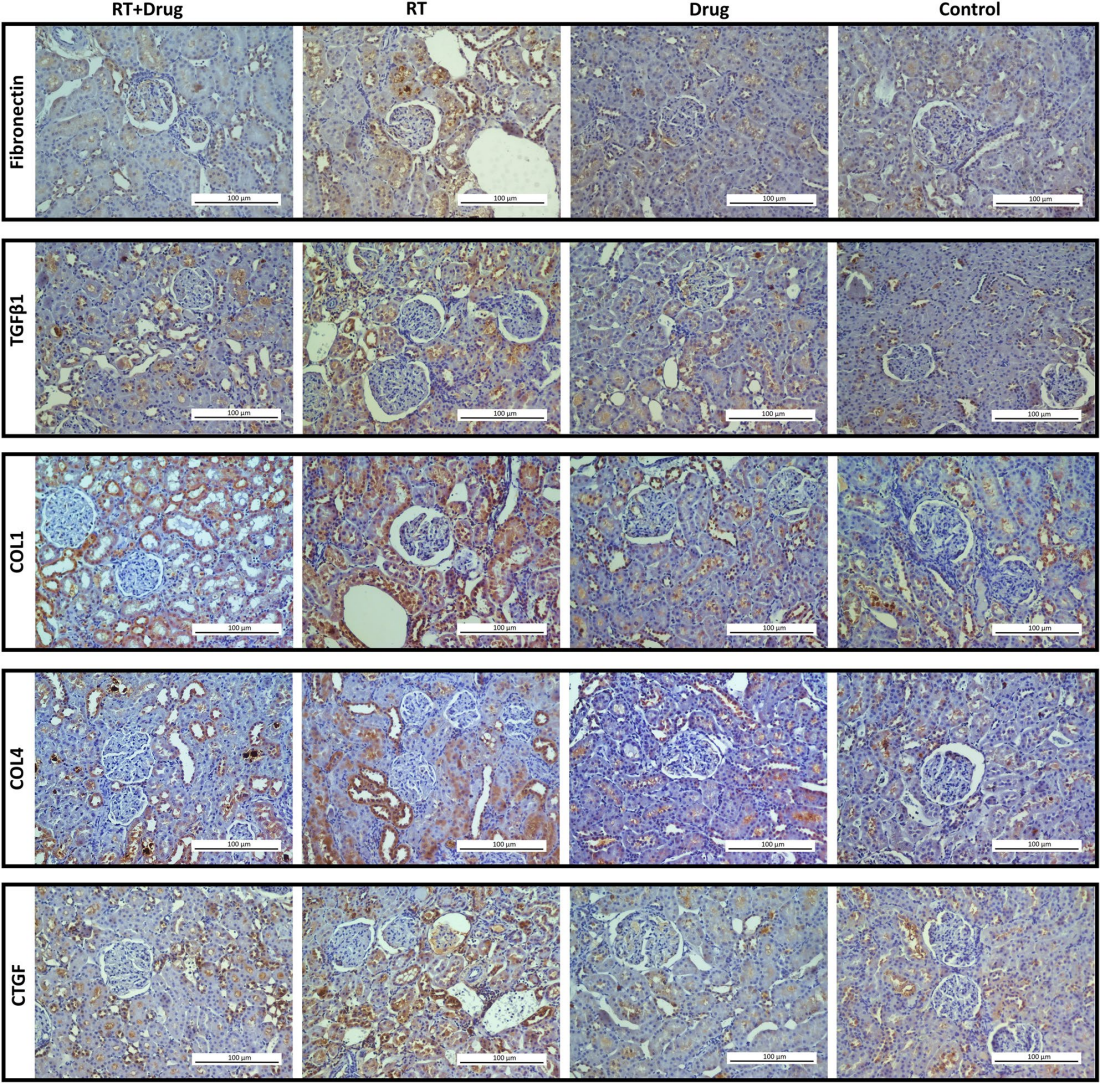
Representative immunohistochemical staining images of renal tissues from the experimental groups (DAB-H&E) (200X).

**Figure 5:**
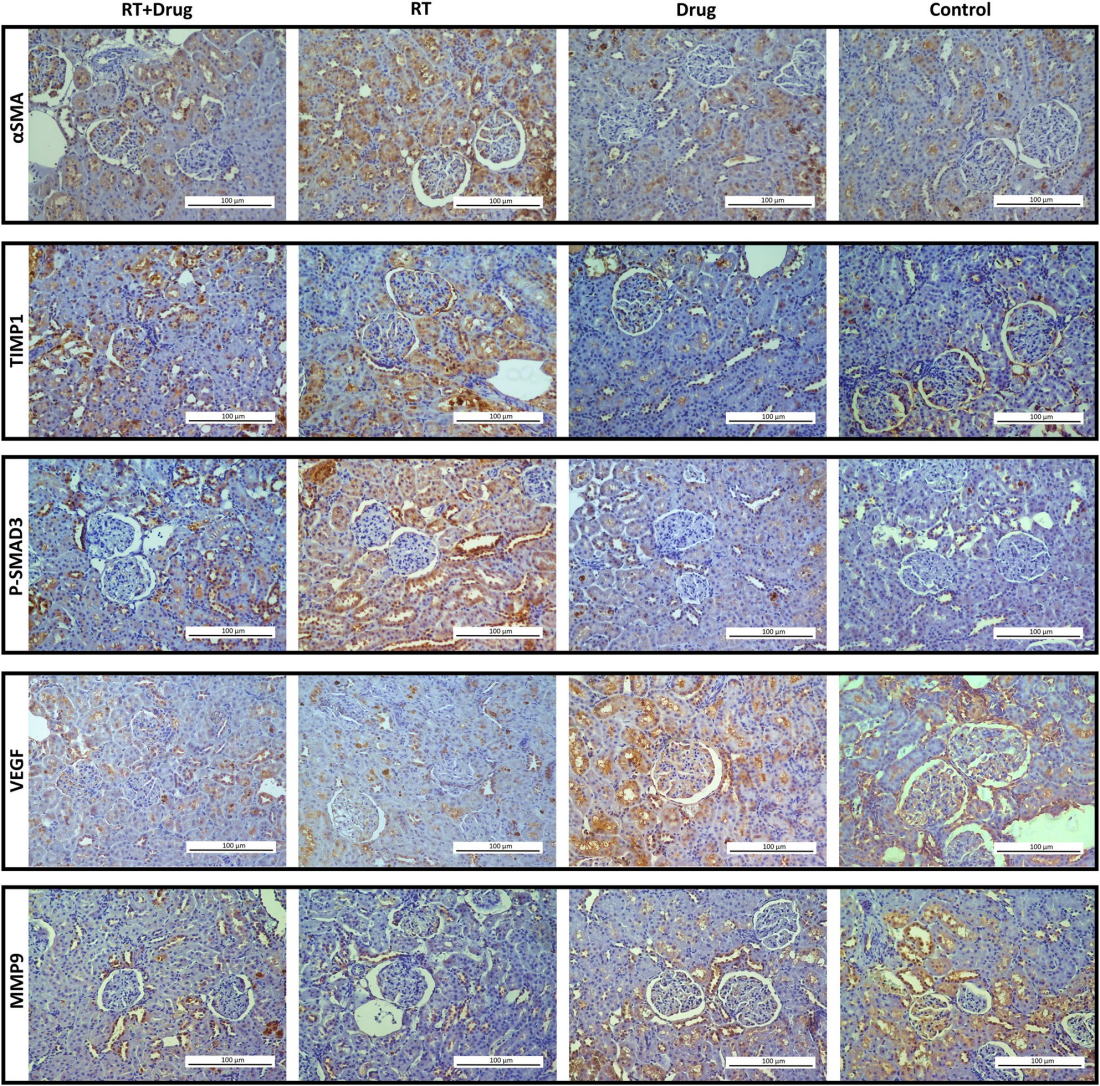
Representative immunohistochemical staining images of renal tissues from the experimental groups (DAB-H&E) (200X).

**Figure 6:**
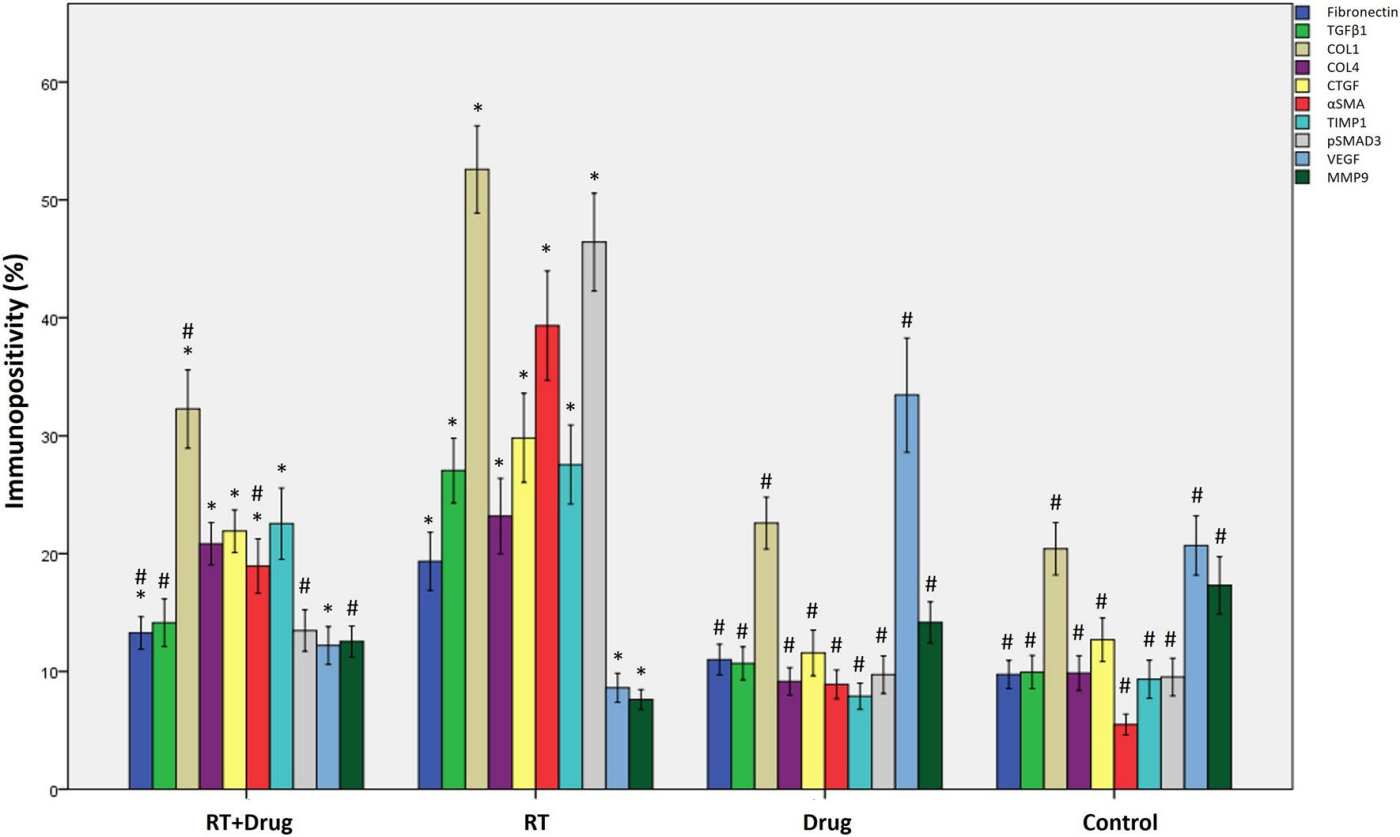
Immune staining ratios for Fibronectin, TGFβ1, COL1, COL4, p-SMAD3, CTGF, αSMA, VEGF, MMP9, and TIMP1 antibodies in renal tissues of all groups (*: significant compared to the control group, #: significant compared to the RT group, *p* < 0.05) (*n* = 36 for each group).

Although the drug-only group did not show significant differences in immunostaining ratio for all markers compared to the control group (*p* > 0.05), the RT-only group showed statistically significant differences compared to the control group (*p* < 0.001). With the exception of VEGF and MMP9, the immunostaining values of the all markers in the RT-only group showed an increase compared to the control group, while a decrease was observed for VEGF and MMP9 (Figs. 4, 5 and 6) (Table 6).

**Table 6:** The pairwise comparison of groups (Immunohistochemistry).

Groups	*p*-value*									
	FN	TGFβ1	COL1	COL4	CTGF	αSMA	TIMP1	p-SMAD3	VEGF	MMP9
Control vs. Drug	1.000	1.000	1.000	1.000	1.000	0.102	1.000	1.000	0.070	0.851
Control vs. RT	** < 0.001**	** < 0.001**	** < 0.001**	** < 0.001**	** < 0.001**	** < 0.001**	** < 0.001**	** < 0.001**	** < 0.001**	** < 0.001**
Control vs. RT + drug	**0.009**	0.067	** < 0.001**	** < 0.001**	** < 0.001**	** < 0.001**	** < 0.001**	0.119	**0.001**	0.089
RT vs. RT + drug	**0.013**	** < 0.001**	** < 0.001**	1.000	0.379	**0.004**	1.000	** < 0.001**	0.204	** < 0.001**
RT vs. Drug	** < 0.001**	** < 0.001**	** < 0.001**	** < 0.001**	** < 0.001**	** < 0.001**	** < 0.001**	** < 0.001**	**0.001**	** < 0.001**
RT + drug vs. Drug	0.276	0.266	**0.005**	** < 0.001**	** < 0.001**	** < 0.001**	** < 0.001**	0.124	** < 0.001**	1.000

Significant values are indicated in bold. * Post-hoc tests with Bonferroni correction following the Kruskal–Wallis test.

The treatment group showed significant increases in the immunostaining ratios of FN, COL1, COL4, CTGF, αSMA, and TIMP1 compared to the control group (*p* = 0.009; < 0.001; < 0.001; < 0.001; < 0.001; < 0.001, respectively). However, this increase was not statistically significant for TGFβ1 and p-SMAD3 (*p* = 0.067; 0.119, respectively). A statistically significant decrease was observed in the VEGF immunostaining ratio in the treatment group compared to the control group (*p* = 0.001), but this decrease was not statistically significant for MMP9 (*p* = 0.089) (Figs. 4, 5 and 6) (Table 6).

Compared to the RT-only group, the treatment group demonstrated a statistically significant decrease in immunostaining ratios of FN, TGFβ1, COL1, αSMA, and p-SMAD3 (*p* = 0.013; < 0.001; < 0.001; 0.004; < 0.001, respectively). On the contrary, the expression level of MMP9 showed a statistically significant increase in the treatment group compared to the RT-only group (*p* < 0.001). The immunostaining ratios of COL4, CTGF, and TIMP1 between the treatment and RT-only groups remained statistically insignificant, despite a relative decrease compared to the RT-only group (p = 1.000; 0.379; 1.000, respectively) (Figs. 4, 5 and 6) (Table 6).

### TUNEL assay

**Figure 7:**
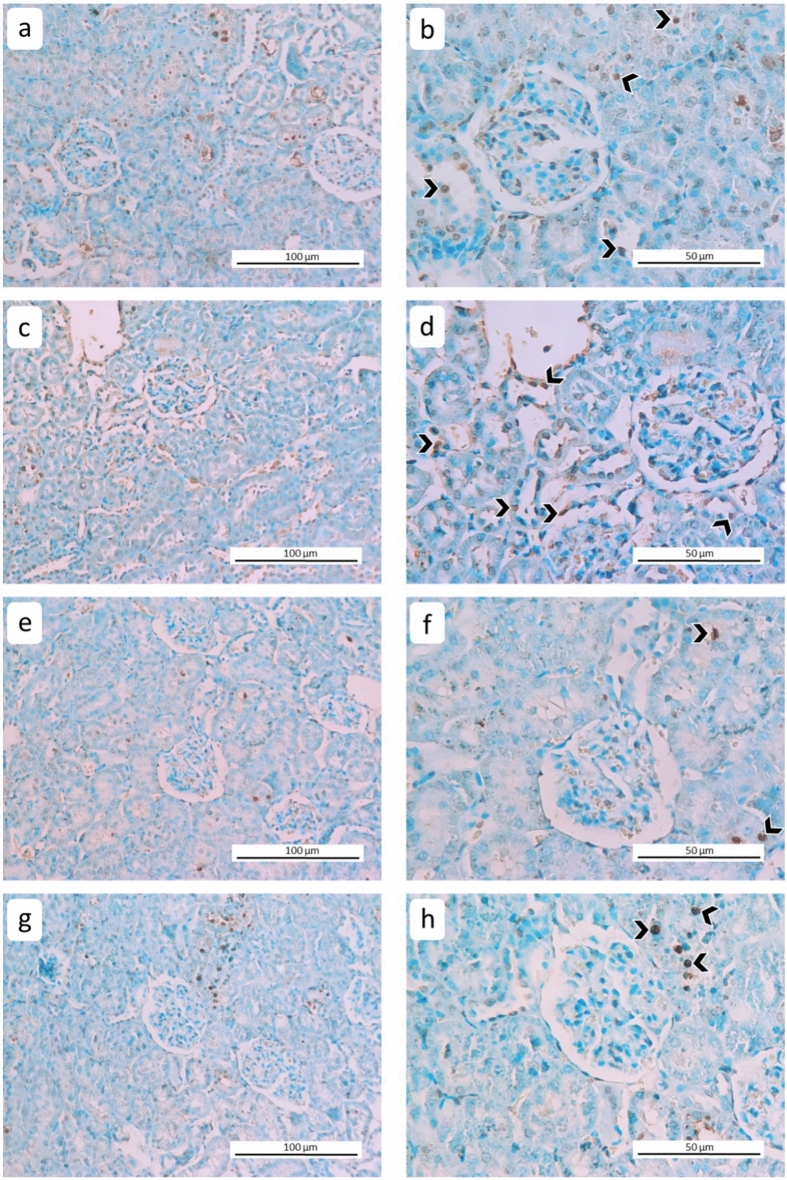
Representative photomicrographs of apoptotic cells obtained by the TUNEL method in renal tissues of the experimental groups. (**a**,**b**) RT + Drug, (**c**,**d**) RT, (**e**,**f**) Drug, (**g**,**h**) control (arrowheads indicate TUNEL-positive cells) (DAB-methyl green) (left column: 200X, right column: 400X).

**Figure 8:**
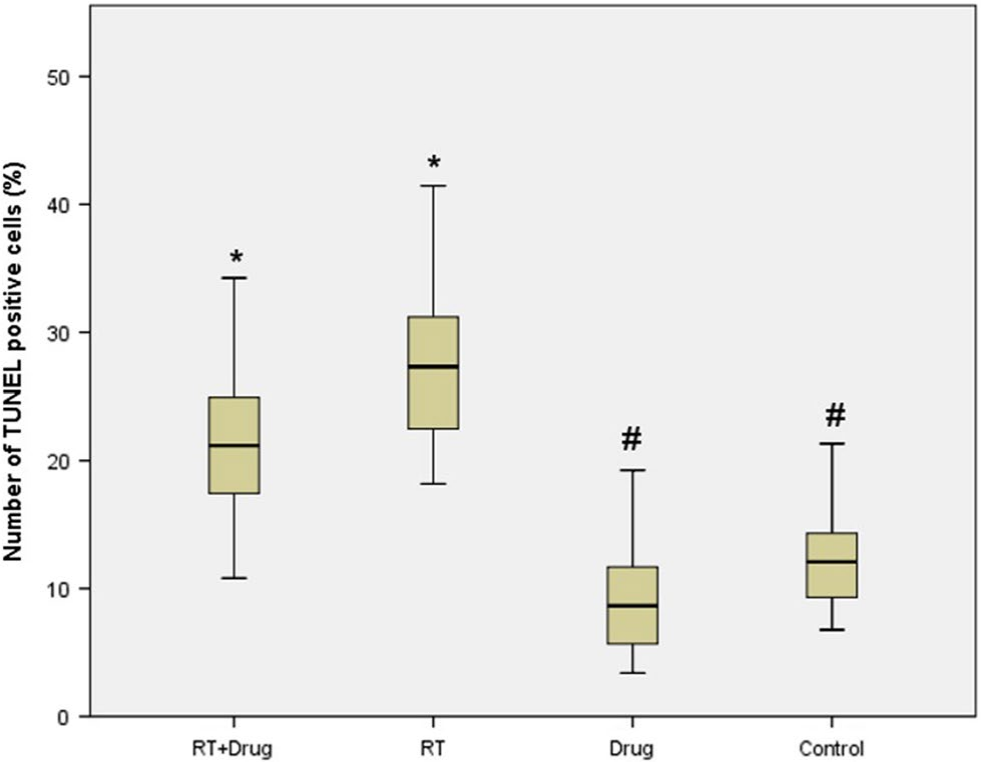
The rates of TUNEL-positive cells in renal tissues of all groups (*: significant compared to the control group, #: significant compared to the RT group, *p* < 0.05) (*n* = 36 for each group).

No differences were observed between the control and drug-only groups (p = 0.423), while the RT-only and RT + drug cohorts showed a statistically significant increase in TUNEL-positive cells compared to the control group (*p* < 0.001). Although the difference between the RT-only group and the treatment group was not statistically significant (*p* = 0.213), the number of TUNEL positive cells decreased in the treatment group. A statistically significant increase was observed in the RT-only and RT + drug groups compared to the drug-only group (*p* < 0.001) (Figs. 7 and 8) (Table 7).

**Table 7:** The pairwise comparison of groups (TUNEL).

Groups	*p*-value*
Control vs. Drug	0.423
Control vs. RT	** < 0.001**
Control vs. RT + drug	** < 0.001**
RT vs. RT + drug	0.213
RT vs. Drug	** < 0.001**
RT + drug vs. Drug	** < 0.001**

Significant values are indicated in bold. * Post-hoc tests with Bonferroni correction following the Kruskal–Wallis test.

## Discussion

The kidneys are dose-limiting variables for radiotherapy in gastrointestinal, gynecologic, and genitourinary cancers, as well as TBI, because of their proximity to radiation fields. Despite efforts to reduce radiation exposure to the kidneys during RT planning, the incidence of RIRF and subsequent chronic renal failure in the literature remains between 15 and 50%^2^. Although substantial kidney dose reduction has been achieved in recent years due to improved RT techniques, a suitable radioprotective agent for the kidneys has yet to be identified. However, based on previous data demonstrating the effectiveness of this treatment in mitigating radiation-induced skin, breast, and pulmonary fibrosis in clinical studies, our findings show that the combination of PTX and vitamin E is beneficial in preventing RIRF in rats and may be extrapolated to humans as well.

The complex mechanisms underlying RIRF have been extensively studied. SMAD, TGF-β, and NF-κB are among the intracellular molecular pathways implicated in the formation of fibrotic tissue following radiation exposure^21^. RT is believed to induce autocrine and paracrine interactions inside renal cells via cytokines and biological mediators. This interaction causes the overproduction of ECM proteins and a simultaneous decrease in their degradation. These interactions often increase the expression of ECM components, such as fibronectin, collagen 1, collagen 3, and TGF-β^4–7^.

Furthermore, fibroblasts convert into myofibroblasts, secreting α-SMA during fibrosis progression. This activation of interstitial myofibroblasts is considered a precursor of fibrosis in irradiated rat kidneys^22^. Additionally, studies on RIRF have found an increase in TGF-β staining in kidney tubules 8 weeks after exposure to radiation doses ranging from 10 to 20 Gy, indicating a proportional relationship between fibrosis severity and radiation dose and duration^7^.

Consistent with the literature, we found a remarkable increase in the expression of α-SMA, TGF-β, TIMP1, fibronectin, collagens 1 and 4, SMAD, and CTGF through immunohistochemical analysis in the RT-only group compared with the control group. These molecular findings were associated with histopathological examinations, which revealed diffuse damage in over 70% of the RT-only group (Table 4). Therefore, our findings support the well-known molecular pathways that cause RIRF.

The interaction between TGF-β activation and the SMAD pathway in radiation-induced fibrosis has been widely studied. TGF-β phosphorylates SMAD2 and SMAD3 receptors through the activation of serine–threonine kinase, forming the SMAD complex. This complex then translocates into the cell nucleus, where it binds to coactivators, such as p300 or Creb, increasing TGF-β transcription. Nonetheless, pentoxifylline inhibits the binding of the SMAD2/3 receptor complex with SMAD4, disrupting TGF-β/SMAD signaling and consequently reducing TGF-β expression^23^.

Our study found that the activated form of SMAD3, p-SMAD3, was upregulated in the RT-only group. However, the following treatment downregulated p-SMAD3 expression. Additionally, the expressions of TGF-β1 and p-SMAD3 were comparable in the control and treatment groups, although the levels in the treatment group were significantly lower than those in the RT-only group. These findings support the role of the TGF-β–SMAD pathways in RIRF and show the efficacy of the PTX and vitamin E combination in modulating this molecular pathway.

VEGF is an essential signaling protein in angiogenesis and vascular permeability. Its role in the progression of renal fibrosis is not yet fully understood. According to Miao et al.^24^, VEGF can suppress the expression of SMAD3 and miR-192, preventing TGF-β-induced epithelial-to-mesenchymal transition (EMT) and alleviating renal fibrosis. Our findings support this, as we found a significant decrease in VEGF expression in the RT-only group compared with that in the control group. This VEGF downregulation may contribute to the profibrotic environment in the irradiated kidney.

Our study showed that combining PTX with vitamin E resulted in a lesser reduction in VEGF expression compared with the RT-only group. In contrast, the drug-only group had higher VEGF levels than the control group, indicating that the drug combination may increase renal VEGF levels. Although this finding did not reach statistical significance (*p* = 0.07), there is a trend toward improving VEGF expression with drug use. This finding suggests that reduced VEGF levels may be associated with the development of RIRF. In support of our findings, Zhou et al.^12^ reported comparable VEGF levels in their study on the effects of PTX on tubulointerstitial fibrosis in obstructive nephropathy. However, it is worth noting that some preclinical studies have found lower VEGF levels in other tissues when using this drug combination^25,26^. Thus, further research is needed to fully understand the mechanisms VEGF contributes to RIRF and the potential therapeutic benefits of targeting this pathway.

In addition, we found that RT-induced TGFβ1 upregulation increased the synthesis of fibrotic markers, such as FN and collagens. However, in our study, the treatment group showed reductions in the expression levels of FN, COL1, and COL4, particularly FN and COL1. CTGF, α-SMA, and TIMP1 expression levels increased in the RT-only groups, consistent with fibrosis, but were shown to be reduced in the treatment groups, particularly for α-SMA. It should be noted that α-SMA is the primary marker for distinguishing differentiated myofibroblasts^22^. Some studies have also reported that macrophages play a role in lung tissue fibrosis by activating α-SMA and promoting the synthesis of COL1^27,28^. Therefore, α-SMA, FN, and COL1 are notable predictors of tissue profibrotic processes, consistent with previous research and our findings.

Furthermore, the antifibrotic marker MMP9 expression was significantly reduced after RT but increased significantly after treatment, indicating a potential shift away from the fibrotic phenotype. MMP9 expression levels were comparable in the control and treatment groups. These findings of MMP9 are consistent with previous research^29^. In conclusion, our immunohistochemical and histopathological findings support our initial hypothesis.

However, there were no statistically significant differences in creatinine levels across the groups. This lack of difference is due to low nephron dysfunction in the subacute phase and a longer (chronic) experimental duration to observe an increase in serum creatinine levels^30^. Therefore, Ahmad et al.^31^ found that creatinine levels were higher after 20 weeks of renal injury, which they attributed to the extended latency period necessary for radiation nephropathy.

Furthermore, several studies have shown the importance of M2 macrophages in treating renal fibrosis^32^. M2 macrophages have been shown to release high levels of TGF-β1, which contributes to renal fibrosis caused by EMT^32^. STAT6 is also required for M2 macrophage polarization, ECM protein deposition, and myofibroblast differentiation^32^. In addition, renal fibrosis may be reduced using AS1517499, a specific STAT6 inhibitor^32^. This pathway may be helpful in RIRF, which needs further research.

Moreover, another study found that myeloid PTEN deficiency is associated with increased immigration of macrophages, T cells, and fibroblasts to the kidney, resulting in increased kidney injury due to angiotensin-2-related hypertension-induced renal fibrosis^33^. Therefore, the role of PTEN is another topic worth investigating for RIRF.

Our findings suggest that combining PTX and vitamin E may effectively treat radiation-induced fibrosis. This combination reduced diffuse tubular damage by half in rat renal cells. These findings may have considerable implications for improving patients’ quality of life and survival rates undergoing RT.

The main limitation of our study is the inherent differences in drug responses and metabolic processes between human and rat kidneys. Although rats are a commonly used model in biomedical research due to their remarkable biological similarity to humans, it is essential to remember that drug-specific effects and metabolism may differ between species. Such differences could influence the effectiveness of PTX and vitamin E in preventing RIRF in humans. The complex drug absorption, distribution, metabolism, and excretion mechanisms in humans and rats may differ substantially, potentially changing the overall impact and therapeutic outcomes. Therefore, while our findings are promising, they should be interpreted cautiously until further validation in human clinical trials.

## Conclusion

The combination of PTX and vitamin E effectively inhibited the molecular impact of ionizing radiation on renal cells and reduced radiation-induced kidney fibrosis. Future research should confirm these findings in a clinical context, focusing on evaluating the safety, efficacy, and optimal dosage of the PTX and vitamin E combination treatment for patients undergoing RT. Furthermore, given our findings on the role of VEGF in RIRF, further research into the therapeutic effects of targeting this pathway is necessary.

## Data Availability

The data that supports the findings of this study are available on the request from corresponding author, V.D.
